# Management of Anxiety in Parkinson's Disease

**DOI:** 10.1002/mdc3.70144

**Published:** 2025-06-26

**Authors:** Alex J. Berry, Harry Costello, Silvia Jesús, Gary Price, Ashwani Jha

**Affiliations:** ^1^ Department of Neuropsychiatry National Hospital for Neurology and Neurosurgery, Queen Square London UK; ^2^ Division of Psychiatry University College London London United Kingdom; ^3^ Unidad de Trastornos del Movimiento, Servicio de Neurología Instituto de Biomedicina de Sevilla, Hospital Universitario Virgen del Rocío Seville Spain; ^4^ UCL Queen Square Institute of Neurology, University College London London UK

**Keywords:** anxiety, Parkinson's disease, non‐motor, neuropsychiatry, meta‐analysis

## Abstract

**Background:**

Anxiety is a common, distressing, hard‐to‐diagnose and hard‐to‐treat symptom in Parkinson's disease. No formal guidelines exist to assist management.

**Objective:**

We provide a pragmatic guide to detecting and managing anxiety in Parkinson's disease.

**Methods:**

In this educational review, we describe the phenomenology, diagnostic challenges, hypothesized neurobiology, and the rationale for treatments for anxiety in Parkinson's disease. We review the major drug‐classes and non‐pharmacological treatment approaches in current use. We also present a meta‐analysis of pharmacological treatments of anxiety derived from previously published systematic reviews of RCTs for depression in Parkinson's disease in which anxiety was a secondary outcome.

**Results:**

In our meta‐analysis, anxiolytic medications showed a moderate overall anxiolytic effect compared to placebo (standardized mean difference: −0.45 [95% CI: −0.78, −0.12], p = 0.02). There were significant limitations with the studies included in this meta‐analysis, with studies generally having small cohort sizes, and each specific pharmacotherapy was not studied in more than one randomized control trial. We also describe a pragmatic algorithm for individualized pharmacological management, based on our own experience of selecting treatments to optimize side‐effect profile and treatment of comorbid symptoms.

**Conclusions:**

Detecting and treating anxiety likely benefits people with Parkinson's disease, though the current evidence‐base for specific treatments or specific pharmacotherapy remains limited. Further work is needed to investigate the different evidence‐based approaches for managing anxiety in Parkinson's disease.

Anxiety disorders occur in at least one‐third of patients living with Parkinson's disease (PD)^1^ and are a primary predictor of health‐related quality of life, causing significant disability and carer stress.^2^, ^3^ A recent survey of over 1000 PD patients identified anxiety as one of their top three unmet needs and a research priority.^4^ However, despite anxiety being common, disabling, and a source of distress to both patients and their families, recent reviews have found insufficient evidence to inform formal guidelines on pharmacological management^5^ and studies have shown it is frequently missed by clinicians.^6^ In this review we summarize what is currently known about the phenomenology, epidemiology and pathogenesis of anxiety in Parkinson's, and provide pragmatic advice on its management.

## The Clinical Presentation and Phenomenology of Anxiety in Parkinson's Disease

Anxiety exists on a spectrum from a normal, healthy, response to threatening or stressful situations, to a debilitating and disabling disorder that can be defined as an excessive feeling of worry, apprehension or unease. The several major sub‐categories of anxiety disorder defined by the *Diagnostic and Statistical Manual of Mental Disorders V (DSM‐V)* and the *International Classification of Diseases 11* (*ICD‐11*) largely converge in the definitions. Diagnostic criteria of anxiety incorporate physical, behavioral, emotional and cognitive components such as trembling, restlessness, excessive worry/catastrophizing, and avoidance.

In PD, generalized anxiety disorder, social anxiety and “anxiety not otherwise specified” are the most prevalent anxiety disorder subtypes.^1^ A recent qualitative study exploring patient experience of anxiety in PD using semi‐structured interviews found that anxiety was often linked by patients to other PD symptoms. Anxiety was described as “coming in waves alongside other motor symptoms” or “perceived as a reaction to them (motor symptoms).”^7^ Almost all patients reported prominent somatic symptoms including internal shaking, shallow breathing, nausea, and changes in body temperature. Psychological aspects of anxiety raised by patients focused on uncertainty around disease progression and future disability, including fear of death or becoming resistant to medication.^7^

Defining and diagnosing anxiety in PD is difficult because it does not neatly fit into existing DSM‐V and ICD‐11. There are a number of challenges that need to be considered.

Firstly, traditional symptoms of anxiety such as autonomic hyperactivity, restlessness, impaired concentration, insomnia and fatigue,^8^ are also common symptoms of PD and common side‐effects of anti‐parkinsonism drug treatment. On the one hand, anxiety can be missed in PD, for example, when patients with anxiety perceive and communicate their symptoms in terms of motor worsening^3^ leading to anxiety‐induced “pseudo‐parkinsonism.”^9^ If overlooked, this can lead to overmedication with dopaminergic therapies, in an attempt to improve the perceived motor symptoms, increasing the risk of dopamine dysregulation syndrome. On the other hand, symptoms of PD can be mis‐attributed to anxiety. For example, akathisia in PD—thought to be related to reduced central dopamine availability—is experienced as an uneasy sense of “inner tremor,” “inner tension,” and restlessness and can be easily mis‐attributed to anxiety.

Secondly, unlike in primary anxiety disorders, anxiety in PD can fluctuate rapidly over hours. This is traditionally thought to be due to fluctuating absorption of levodopa and nonmotor “off” periods, but the reality is much more complex with a majority of patients not noting any relation between medication doses and anxiety, and those that do noting associations not only with “off” periods, but also with “on” and dyskinetic periods.^3^ Patients also often report a negative cycle of fear of impending “off” states, somatic symptoms (including breathlessness and dizziness), and catastrophizing fears of falling leading to agoraphobia.

Thirdly, anxiety disorders in PD are often associated with an increased incidence of overlapping neurological and psychiatric conditions including restless leg syndrome.^10^ chronic pain,^11^ gait freezing,^3^, ^12^, ^13^, ^14^ falls and most notably, depression.^15^, ^16^ While depression and anxiety disorders can occur independently in PD, they frequently coexist. Co‐morbid depression and anxiety is reported in 19.3% of PD patients.^15^ This high degree of co‐morbidity has led to calls for depression and anxiety to no longer be treated as distinct phenomena. Supporting this, a latent class analysis of depression and anxiety related subtypes in over 500 PD patients identified two clinical phenotypes of depression in PD: one characterized as “anxious‐depressed” and the other “depressed.”^16^ This relationship between depression and anxiety may have important implications for treatment and measurement. Studies have shown that depressive symptoms explain over 40% of the variance in anxiety symptom scales in PD patients.^17^ Additionally, in non‐PD populations, patients with major depression and high anxiety (anxious depression) tend to have poorer outcomes than those with depression alone.^18^ However, no study has examined the effect of co‐morbid anxiety of treatment outcomes in PD. There is also an increased risk of PD‐related anxiety in those with pre‐existing avoidance behaviors and “catastrophising” cognitive styles.^19^, ^20^

Taken together, these complexities can result in diagnostic uncertainty for clinicians, resulting in missed diagnoses during time‐limited consultations.^3^, ^9^

## Use of Symptom Rating Scales in Parkinson's Disease Anxiety

The overlap between the autonomic features of anxiety disorders and non‐anxiety‐mediated symptoms of PD presents obvious problems when interpreting traditional anxiety disorder rating scales.^21^

Factor analysis of systematic anxiety measures (such as the Beck Anxiety Inventory) have found dissociable symptom dimensions of anxiety in PD, which differentiate this syndrome when compared to anxiety disorders observed in the general population.^16^, ^22^ This has contributed to the development of new PD‐specific measures such as the Parkinson's Anxiety Scale (PAS). In Table 1 we summarize the commonly used scales to capture anxiety symptom severity in PD.^21^, ^22^, ^23^, ^24^, ^25^, ^26^, ^27^, ^28^, ^29^, ^30^

**TABLE 1:** Summary of anxiety rating scales in Parkinson's disease.

Scale	Description
Parkinson Anxiety Scale (PAS)^22^	Validated in PD, divided into sections screening for “persisting anxiety,” “episodic anxiety” and “avoidance behavior,” and rates the severity of symptoms. A score of >14 has been suggested as a “cut off” score for an anxiety disorder. There are both self‐rated and observer‐rated versions of the PAS, and they take 2–5 min to administer.
Geriatric Anxiety Inventory (GAI)^23^	A 20‐item self‐report measure of severity of anxiety symptoms. The scale excludes the inclusion of somatic symptoms of anxiety which overlap with PD motor features, making it a useful scale in PD
Non‐Motor Symptoms of Parkinson's disease scale (NMSS)^24^	Clinician‐based scale. Validated in PD and can screen for a multitude of other non‐motor symptoms. Questions are weighted towards depression and apathy rather than anxiety specifically.
Neuropsychiatric Inventory (NPI)^25^	Clinician‐based scale, which can be administered to careers, and may be helpful in identifying problems with caregiver burden. Is validated for use in dementia.
State–Trait Anxiety Inventory (STAI)^26^	Self‐assessment Likert scale, where a cut off >40 has been proposed to detect clinically significant anxiety in the general population
Liebowitz Social Anxiety Scale^27^	Self‐assessment tool using a Likert scale to rate fear and avoidance of several scenarios that may provoke anxiety. This scale has been used by some study groups to assess for presence of anxiety symptoms in PD.
Hospital Anxiety and Depression Rating Scale (HADS)^28^	Self‐assessment symptom severity scale, which has the advantage of screening for depressive symptoms too. Scoring >11 is felt to represent the presence of clinically significant anxiety.
Hamilton Anxiety Rating Scale (HAM‐A)^29^	Clinician‐administered questionnaire. Focused towards detection of generalized anxiety symptoms (rather than panic attacks, or phobic anxiety brought on by particular scenarios)
Beck Anxiety Inventory (BAI)^30^	Self‐assessment scale, focused towards the assessment of panic symptoms predominantly

## Epidemiology of Anxiety in Parkinson's Disease

Reported prevalence rates of anxiety in PD vary widely (6–55%) but the average point prevalence across studies is 31%.^1^ Risk factors for the development of PD anxiety include greater disability, female sex, younger age‐at‐onset, higher levodopa‐daily equivalent doses, insomnia,^31^ prior history of an anxiety disorder^11^ and the presence of motor fluctuations.^11^, ^12^, ^32^ Patients with genetic PD due to mutations in the *SNCA*,^33^ *GBA* and *LRRK2* ^34^ may have an increased risk of developing anxiety disorders (and mood disorders overall) compared to idiopathic PD.

Whether anxiety disorders are a prognostic risk factor or an early prodromal symptom in the development of PD remains unclear. Phobic anxiety traits^11^, ^16^, ^32^ and the prescription of anxiolytic medications have been reported to be associated with an increased risk of developing PD.^32^, ^35^, ^36^ A significant proportion of those who develop PD anxiety, however, develop this after the onset of PD.^16^

## Pathogenesis of Anxiety in Parkinson's Disease

Despite major advances in our neurobiological understanding of PD the biological mechanisms underlying anxiety remain poorly understood.

### Monoamine System Dysfunction

Parkinson's disease is associated with the degeneration of multiple brainstem nuclei that produce monoamine neurotransmitters. Whilst reduced levels of dopamine—as produced by the substantia nigra pars compacta—is classically associated with motor impairment, reduced production of dopamine by the ventral tegmental area, reduced production of serotonin by the raphe nuclei, and reduced production of noradrenaline by the locus coeruleus have been associated with anxiety in PD. The psychiatric consequences of dopaminergic neurodegeneration are thought to be mediated by the subsequent dysfunction of limbic cortico‐striatal‐thalamocortical circuits.^37^, ^38^, ^39^ Patients with PD and anxiety have reduced binding rate of striatal dopamine transporter (DaT) even after controlling for motor symptom severity.^40^ Reduced serotonin transporter binding in the subgenual and dorsal anterior cingulate cortex is also associated with increased anxiety symptoms in PD.^41^ A recent PET study that combined ^11^C‐yohimibine (a radioligand that binds to α2 adrenergic receptors) with neuromelanin sensitive MRI (to delineate the locus coeruleus) found that PD patients with higher anxiety had reduced α2 adrenergic receptors in the putamen, insula and superior temporal gyrus.^42^

Most anxiolytic treatments modulate the monoamine systems in the brain but, to our knowledge, the effect of these treatments on degenerating monoamine systems in Parkinson's has not been studied mechanistically.^43^ This is challenging because of the dynamics and function of monoamine pathways are diverse, complex and interdependent. For example, dopamine is co‐released with and modulated by serotonin within the striatum.^44^ Further understanding of the relative deficits in monoamine function across stages of PD, their interaction, and the relationship with the emergence of anxiety symptoms could potentially guide treatment strategy.

### Fronto‐Amygdala Dysfunction

Animal models of anxiety have identified key roles of the amygdala, bed nucleus of the stria terminalis (BNST), anterior insula and prefrontal cortex (PFC) in anxiety symptoms and response to threat.^45^ Functional MRI studies in patients with anxiety without PD have largely converged on the neural circuitry identified in animal models. Increased activity in the dorsomedial PFC has been associated with pathological anxiety and reduced ability to extinguish fear.^45^, ^46^ Higher trait anxiety is also associated with increased connectivity between the amygdala and mPFC, while heightened sensitivity to unpredictable threats in patients with anxiety has also been associated with increased BNST activation.^45^, ^47^, ^48^ The therapeutic effects of anxiolytic treatment have been proposed to be partly mediated by reduced engagement of this dmPFC‐amygdala circuit. Selective serotonin reuptake inhibitors (SSRIs) result in reduced connectivity between the BNST, and limbic structures.^46^, ^49^ A recent systematic review comprising 18 neuroimaging studies of anxiety in PD reported increased connectivity between the mPFC and amygdala but reduced connectivity between the medial PFC, anterior cingulate cortex and insula.^40^ Across different classes of anxiolytics, modulation of the dynamics of the amygdala‐PFC circuit appears to be a key driver of therapeutic efficacy. Further understanding of the neural mechanisms underlying anxiety in PD is required, however, before circuit‐based interventions such as deep brain stimulation can be used to improve anxiety in PD.

## Management of Anxiety in Parkinson's Disease

There is a lack of empirical evidence from clinical trials for the effective management of anxiety in PD. Most evidence to date is based on secondary outcome measures from trials or derived from treatment effects established in the general population without PD. We review current treatments used in PD anxiety and their existing evidence base below.

### Pharmacological Treatment

**Figure 1:**
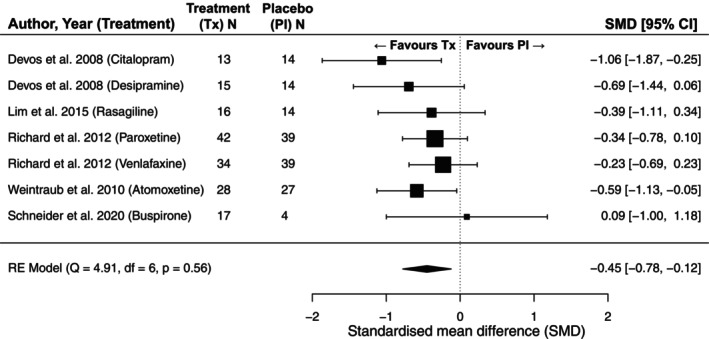
Forest plot of anxiety symptom change with treatment versus placebo in RCTs of pharmacological therapies in PD where anxiety is reported as a secondary outcome measure. The included RCTs were identified from three published systematic reviews of treatments for depression in PD, rather than through an independent systematic review of anxiety treatments. A multilevel meta‐analysis was performed, clustering by study as a random effect to account for multiple treatment arms from two included studies.

A diverse array of pharmacological therapies have been used to manage PD anxiety. However, no randomized controlled trial (RCT) has been conducted where PD anxiety is a primary outcome. We, therefore, conducted the first meta‐analysis of the efficacy of pharmacological therapies for PD anxiety including RCTs where an anxiety measure was reported as a secondary outcome measure (Fig. 1, see supplement for meta‐analysis methods). We identified RCTs by using existing recent systematic reviews^50^, ^51^, ^52^ of anti‐depressants and other therapies in PD and extracted secondary anxiety outcome measures from included studies where reported. Our analysis includes seven medications consisting of five drug classes (SSRIs, serotonin and noradrenaline reuptake inhibitors (SNRIs)), monoamine oxidase type‐B (MAO‐B) inhibitors, noradrenaline reuptake inhibitors (NARIs), 5HT‐1^A^ partial agonist) from five trials (two studies used multiple treatment arms). We found evidence for a moderate therapeutic effect (standardized mean difference (SMD): −0.45 [95% CI: −0.78, −0.12], *p* = 0.02) of pharmacological therapies for anxiety in PD. Analysis of heterogeneity indicated low between‐study variability, with a small between‐study variance component (*σ* ^2^ = 0.015) and a non‐significant *Q*‐test (*Q* (6) = 4.914, *p* = 0.555), suggesting that differences in treatment effects across studies are consistent with random variation rather than true heterogeneity. This finding implies that the included trials yielded relatively consistent effect sizes, reducing the need for subgroup analyses or meta‐regression to account for study‐level differences.

However, our findings should be interpreted with caution due to several limitations. First, all included studies were small in size, and anxiety outcomes were assessed only as secondary measures rather than primary trial endpoints. Second, our approach relied on pre‐existing systematic reviews rather than conducting an independent systematic assessment of anxiety treatments in PD. While these systematic reviews followed rigorous methodologies, they were not designed to comprehensively assess the efficacy of pharmacological treatments for PD anxiety. This raises the possibility that relevant studies—particularly those not included in prior reviews—may have been missed. Finally, there were insufficient studies to meta‐analyze the effects of specific drug classes and no single pharmacological agent was studied in more than one trial, so no RCT result for any one pharmacological agent has been replicated. We also cannot exclude the possibility of there being unpublished trials with negative results. Given these limitations, a dedicated systematic review and meta‐analysis focusing specifically on pharmacological treatments for anxiety in PD is needed to provide a more comprehensive evaluation of treatment efficacy.

### Antidepressants

#### Selective Serotonin Reuptake Inhibitors (SSRIs)

Despite a relative lack of RCT‐level evidence for using SSRIs in anxiety in PD, SSRIs remain the most frequently prescribed medication for PD anxiety.^53^ The most‐studied SSRIs for anxiety disorders in the general population are citalopram, escitalopram, sertraline, fluoxetine, fluvoxamine and paroxetine.^54^ Selecting a specific SSRI is mainly guided by side effect profile since most SSRIs have similar efficacy rates when reviewing RCT evidence from the general population.^54^, ^55^, ^56^

Sertraline (50–100 mg daily) is the best‐tolerated SSRI in the general population and has been associated with higher quality of life scores when examined in trials on PD depression.^57^, ^58^

Paroxetine (30–40 mg daily) failed to demonstrate a significant difference in placebo‐reducing anxiety scores after 12 weeks of treatment.^59^ Citalopram 20 mg daily showed a reduction in anxiety rating scales from baseline, though at 4 weeks post‐initiation no difference was observed when compared to placebo.^60^ A small open‐label study has shown improvement in gait‐freezing with escitalopram and paroxetine in PD.^61^

Importantly, few RCTs have looked at the effects of SSRIs on anxiety in patients over 65 years of age, which limits the generalizability of study findings. Significant side‐effects of all SSRIs include worsening motor features.^60^ There is now increasing awareness of SSRI‐withdrawal syndromes, affecting some patients after abrupt discontinuation.

#### Serotonin and Noradrenaline Reuptake Inhibitors (SNRIs)

Whilst SSRIs are generally better tolerated than SNRIs, though there is also some evidence that SNRIs are more effective in treating anxiety disorders in the general population.^54^, ^55^, ^56^, ^60^ SNRIs such as venlafaxine and duloxetine exert their effects by inhibiting presynaptic membrane serotonin and norepinephrine protein transporters. Few studies have assessed the effectiveness of SNRIs in mood disorders in PD.^61^, ^62^ Both venlafaxine and duloxetine demonstrate effectiveness above placebo in the general population, with slightly more patients achieving remission on venlafaxine than duloxetine.^64^ Contrastingly, a study comparing venlafaxine (mean dose of 121 mg per day) against placebo in PD patients, did not observe reductions in anxiety scores after 12 weeks of treatment.^63^

Duloxetine has been associated with benefits in chronic pain^65^ and gait freezing in PD.^61^ It may be helpful to consider duloxetine when these disorders co‐exist with PD anxiety, given their close association with anxiety.

#### Noradrenaline Reuptake Inhibitors (NARIs)

Atomoxetine is licensed for treatment of attention deficit hyperactivity disorder. Its efficacy as an antidepressant has been investigated in a single study, looking at PD depression cases – where atomoxetine demonstrated benefit on a secondary measure of anxiety compared to placebo.^66^ However, the effect size appeared to be small. Atomoxetine possesses stimulant‐like effects and might be a therapeutic option where anxiety may co‐exist with impulsive behaviors or excessive daytime sleepiness.

#### Tricyclic Antidepressants (TCAs)

Two small placebo randomized controlled trials have compared nortriptyline and desipramine to paroxetine and citalopram, respectively.^59^, ^60^ Both reported tricyclic antidepressants significantly lowered anxiety rating scales (secondary measures).^59^, ^60^ While nortriptyline (at doses of 25–75 mg/day) significantly outperformed both paroxetine and placebo, desipramine (at doses of 75 mg/day) failed to demonstrate superiority over placebo.^59^, ^60^

Although the side‐effect profile of TCAs likely deters prescribers from initiating these, a network meta‐analysis found that TCAs compared favorably to SSRIs in terms of tolerability in PD and suggested they are perhaps more efficacious than SSRIs or SNRIs in treating anxiety.^50^ The desired psychoactive effects of TCAs are achievable at lower doses than in the general population, and lower doses likely reduce the risk of side effects.^50^

#### Buspirone

Buspirone is a 5HT_1A_ partial agonist. An observational study found an anxiolytic effect at a low dose (doses under 60 mg/day). At higher doses of 100 mg daily, buspirone reduced dyskinesia in PD, but anxiety scores increased—possibly relating to Buspirone's D2/D3 antagonist properties at higher doses.^67^ A more recent double‐blind placebo‐controlled RCT showed 1530 mg buspirone was poorly‐tolerated in PD. Over half of patients receiving buspirone experienced worsening motor symptoms, and the tolerability concerns prevented analysis of the effects on anxiety.^68^

#### Mirtazapine

Though frequently prescribed, Mirtazapine is not licensed for use in anxiety disorders in the UK, and its effects on PD anxiety has not been studied. Alongside its antidepressant effect, mirtazapine may help tremor.^69^ Doses of 7.5–15 mg are strongly antihistaminergic and well‐established to help promote sleep, though mirtazapine has also been associated with REM sleep behavior disorder exacerbations.^70^

#### Trazodone

Trazodone is an antidepressant used in the treatment of primary insomnia and anxiety. It lacks affinity to acetylcholine receptors, making it a particularly useful medication for those with PD who may be sensitive to antimuscarinic side effects such as constipation. It has been associated with postural hypotension, QT‐prolongation, and rarely priapism. A single open‐label study, using 25–150 mg daily dosing, reported significant reductions in anxiety in 39 patients with PD after 12 weeks of treatment and improvements in mood and sleep‐rating scales.^71^ Rarely, cases of parkinsonism have been described with trazodone.^72^

#### Bupropion

Bupropion is a dual reuptake inhibitor of dopamine and norepinephrine (NDRI), typically used in nicotine dependence but is known to exert an anxiolytic effect. However, robust evidence for its use is currently lacking, with only a few case reports having evaluated its action on panic disorder in PD.^73^

#### Agomelatine

Agomelatine is an atypical antidepressant that is an agonist at melatonin receptors (MT1 and MT2) and antagonist at the serotonergic 5HT_2C_ receptor (the latter may underly an anxiolytic effect). There is some limited evidence for agomelatine as potentially helpful for low mood and insomnia in PD and some evidence for its use in anxiety disorders in the general population. There is limited evidence to suggest a motor benefit in PD from agomelatine, but no randomized controlled trial data exists in PD anxiety.^74^, ^75^

### Anti‐Parkinsonian Therapies

#### Monoamine Oxidase Inhibitors (MAOIs)

Selective type‐A monoamine oxidase (MAO‐A) inhibitors, such as moclobemide, are primarily used in mood disorders to prevent the catabolism of serotonin and noradrenaline.^76^ In contrast type‐B monoamine oxidase (MAO‐B) inhibitors, such as rasagiline and selegiline, were developed for treatment in PD due to their ability to inhibit dopamine catabolism, thereby increasing striatal dopamine levels.^76^ There is a possible risk of serotonin syndrome in those co‐prescribed SSRIs or SNRIs and MAOIs, but the incidence of this appears to be very low.^77^ No studies to date have investigated MAO‐A inhibitors as treatments for PD anxiety. In contrast, MAO‐B inhibitors are commonly used in PD and cross‐sectional studies have shown their use is associated with a reduced prevalence of anxiety disorders in PD.^11^ However, evidence regarding their use is mixed, with two RCTs of rasagiline; a large trial (n = 192) of rasagiline in combination with an antidepressant for non‐motor symptoms and another smaller trial for fatigue (n = 40), reporting no significant difference versus placebo on secondary anxiety outcome measures.^78^, ^79^ Similarly, use of rasagaline was not associated with reductions in anxiety rating scores in a cohort of patients with prominent freezing of gait.^80^ While no group has studied selegiline or safinamide's effects on PD anxiety, it is tempting to speculate that they may be helpful in some cases where anxiety is associated with prominent off‐periods.^81^ Longitudinal studies in depression have shown that MAO‐B inhibitors improve both depressive and motivation symptoms but this is dependent on disease severity and striatal dopaminergic neurodegeneration.^82^ Further trials and longitudinal studies are needed to understand the therapeutic role of MAOIs in PD anxiety.

#### Continuous Dopaminergic Stimulation

There is a growing evidence base to support the use of continuous dopaminergic stimulation (such as an apomorphine subcutaneous pump, levodopa/carbidopa intestinal gel (LCIG) infusion Duodopa®, or foslevodopa/foscarbidopa subcutaneous pump) in improving quality of life for people with PD. Use of these preparations may lead to a reduction in anxiety symptoms and motor fluctuations.^83^ However, these studies often have small sample sizes (typically under 30 participants), and results on the effectiveness of these treatments on non‐motor symptoms are mixed.

### Other Pharmacological Therapies

#### Benzodiazepines

Benzodiazepines can rapidly relieve anxiety symptoms and may outperform several other anxiolytic treatments.^84^ We are aware of only one study investigating the use of bromazepam in treating PD anxiety, which reported benefits in both anxiety and tremor, relative to placebo.^85^ Due to the risk of side effects, including dependence‐formation, falls and worsening cognitive impairment, benzodiazepines are rarely the first choice for treating anxiety. Nevertheless, benzodiazepines are still widely prescribed to treat PD anxiety, which may reflect a degree of partial‐ or non‐response observed with SSRIs and SNRI treatments in PD anxiety.^53^ Prescribing benzodiazepines should arguably be avoided, if possible, in patients with a history of substance dependence. There is a need for responsible prescribing and careful monitoring of benzodiazepine in PD anxiety, but these can nevertheless be highly effective treatments for difficult‐to‐treat cases.

#### Quetiapine

Quetiapine is an atypical antipsychotic, possessing a lower tendency than most antipsychotics to antagonize D2 dopamine receptors, meaning it can be used safely in PD (at doses of under 200 mg per day but typically starting much lower eg, 12.5 mg per day).^86^ Partial agonism of 5HT_1A_ receptors and norepinephrine reuptake inhibition are thought to underlie the anxiolytic effect of quetiapine.^87^ Antipsychotics have been used as both monotherapies, and augmenting agents alongside antidepressants, in treating anxiety in the general population.^88^, ^89^ Anxiolytic doses in PD may also be significantly lower than those used in the general population.^90^ It is important to note that treatment of anxiety remains an off‐license use of quetiapine. The most frequently encountered side effects are sedation, hypotension, weight gain, dylipidaemia and impaired glucose tolerance. Quetiapine is liable to increase the QT interval length, so ECG monitoring is recommended before and during treatment.

#### Pregabalin

Doses of 200 mg daily have been reported as effective in anxiety disorders associated with neurodegenerative disorders,^91^ though no study has specifically looked at the use of pregabalin in PD anxiety. There are reports of pregabalin being associated with worsening Parkinsonism.^92^ Pregabalin may be helpful when restless leg syndrome is comorbid with an anxiety disorder. Consideration should be given to pregabalin's liability to form dependence. A prescribing approach similar to that taken when prescribing benzodiazepines may be helpful—with careful monitoring, and exercise of caution if a patient has a history of substance dependence.

#### Cannabinoids

Cannabinoids have attracted interest as novel (and potentially well‐tolerated) anxiolytic agents in PD. Cannabidiol is a negative allosteric modulator at CB1 and CB2 cannabinoid receptors and a partial agonist at 5HT1A receptors. Cannabidiol also inhibits the activity of the enzyme fatty acid amide hydrolase (FAAH), an enzyme responsible for catabolism of endocannabinoids.^93^, ^94^ Cannabidiol at 300 mg daily has previously been reported to increase quality of life relative to placebo in PD, though notably not in “emotional wellbeing” measures.^95^ Nabilone, a synthetic analogue of tetrahydrocannabinol and partial agonist of CB1 and CB2 receptors, reduced anxiety subscales scores on MDS‐Unified Parkinson's Disease Rating Scale (MDS‐UPDRS) (but not on other secondary measures of anxiety) in a small double‐blinded trial among PD patients without major psychiatric disorders.^96^ Further research is needed before meaningful recommendations can be made about cannabinoids.

### Non‐pharmacological Therapies

#### Psychological Therapies

Cognitive behavioral therapy (CBT) is considered the gold standard of psychotherapy treatments for anxiety disorders in the general population.^103^ However, there is a mixed evidence base for CBT for PD anxiety, with significant methodological flaws. Studies to date have been small, often lack appropriate control groups, and several do not show beneficial effects of CBT.^97^ One study reported a significant reduction of anxiety symptoms following CBT compared to a “waitlist” control (use of waitlist control groups have been shown to inflate apparent therapeutic effect sizes of active interventions such as CBT).^98^, ^99^ Another small (*n* = 16) pilot study of CBT reported a benefit on depression but no effect on anxiety.^97^ A larger evidence base, though with many of the same limitations, exists for depression in PD which does show efficacy in improving mood symptoms.

Other psychological therapies trialed in PD anxiety include mindfulness‐based therapies. The largest (n‐138) randomized controlled trial of mindfulness‐based therapy to date reported significant benefits of a mindfulness yoga program versus stretching/resistance exercises on anxiety symptoms.^100^ However, this study only included PD patients with minimal disability that were independently mobile. Other small trials of mindfulness therapies have shown mixed findings.^101^, ^102^

A recent systematic review of psychotherapies for anxiety in PD found that most randomized control trials examined anxiety as a secondary measure, but there was evidence‐base for anxiety reduction using CBT approaches, though there were mixed results for mindfulness‐based therapies.^103^ Further trials exploring the efficacy “third‐wave” psychotherapies such as Acceptance Commitment Therapy are needed.

#### Physiotherapy

The evidence base for physiotherapy in PD anxiety appears mixed. A systematic review including 20 studies evaluating non‐motor symptom improvement reported that increased physical activity positively affected depression, anxiety, apathy, fatigue, sleep and cognition.^104^ However, a more recent tablet‐based physiotherapy training program found no significant effect on anxiety symptoms at 9 months in 137 PD patients.^105^

#### Transcranial Magnetic Stimulation (TMS) and Electroconvulsive Therapy (ECT)

Low‐frequency repetitive TMS has been reported to be associated with benefits in motor function in PD, though the effectiveness of TMS in anxiety is yet to be studied.^106^ Transcranial direct current stimulation (tDCS) has not been studied in PD anxiety.

Successful use of electroconvulsive therapy has been described in several case reports for cases of severe anxiety refractory to medical treatment (occurring with depression or psychosis).^107^, ^108^ There are case reports of ECT being successfully administered to patients with deep brain stimulators in situ.^108^

#### Deep Brain Stimulation (DBS)

A single meta‐analysis of PD and DBS reported that stimulation at the globus pallidus interna was associated with modest improvements in anxiety rating scale scores, which persisted for over 6 months and was not found in those who underwent subthalamic nucleus stimulation. However, the included studies were highly heterogeneous, so caution is required when interpreting this result.^109^ However, more recent studies have reported improved anxiety 6‐months postoperatively following subthalamic nucleus (STN) stimulation, possibly related to relatively‐ventral electrode placement within the STN.^110^

Withdrawal of dopamine agonists following the insertion of DBS may also produce anxiety states, so clinicians should be alert to the risks of dopamine agonist withdrawal syndrome (DAWS).

## Conclusion

This article reviews the clinical presentation, epidemiology, potential mechanisms, and management of anxiety in PD. We also highlight the urgent need for further trials of therapeutic interventions in this relatively neglected patient group.

The available evidence regarding treatment for PD anxiety remains limited, often based on extrapolations from trials for anxiety in the general population or studies primarily investigating depression in PD. However, anxiety in PD probably has distinct underlying mechanisms to anxiety in the general population requiring tailored therapeutic interventions and further studies are needed. Investigating how anxiety in PD develops could also provide mechanistic insights into anxiety in general and help to identify novel therapeutic targets.

**Figure 2:**
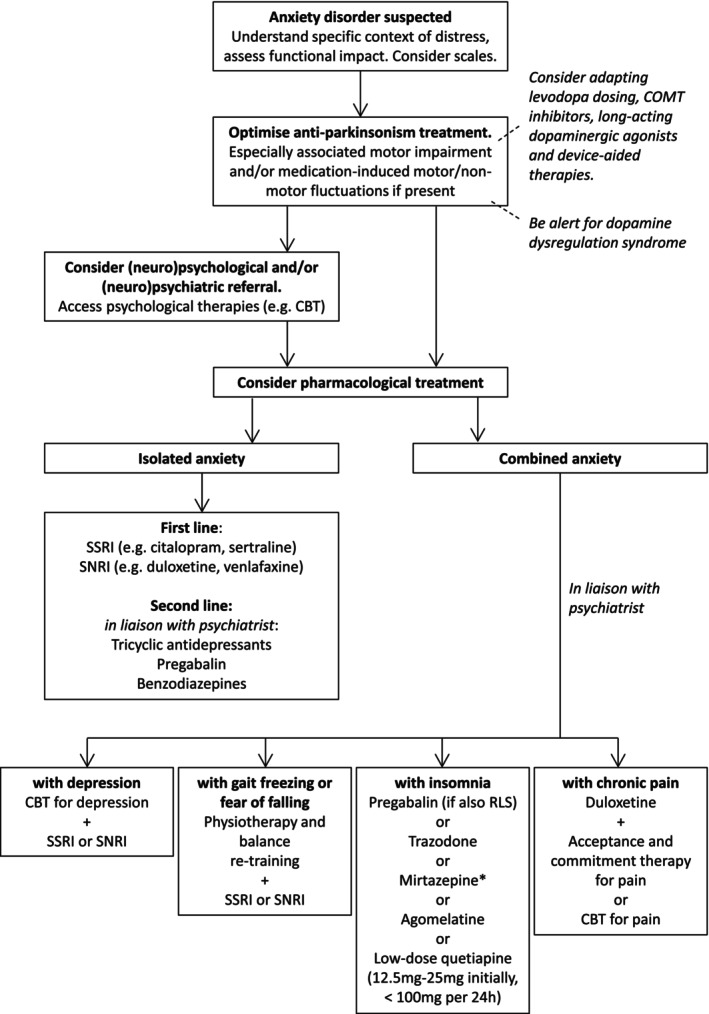
A proposed algorithm for the management of anxiety in Parkinson's disease. Diagnosis of Parkinson's disease anxiety can be supported with the use of scales such as the Geriatric Anxiety Inventory (GAI), Parkinson's Anxiety Scale (PAS), and Neuropsychiatric Inventory (NPI). Use of second‐line of combinations of agents will typically require the support of a (neuro)psychiatrist. ACT, Acceptance and Commitment Therapyl; CBT, Cognitive Behavioral Therapy; COMT, Catechol‐O‐methyltransferase; GAI, Geriatric Anxiety Inventory; NPI, neuropsychiatric inventory; PAS, Parkinson's Anxiety Scale; RLS, Restless Leg Syndrome; SNRI, Serotonin and Noradrenaline Reuptake Inhibitor; SSRI, Selective Serotonin Reuptake Inhibitor.

In the absence of formal guidelines, we present a meta‐analysis of pharmacological treatments of anxiety—based on secondary outcomes in RCTs (Fig. 1)—and a pragmatic algorithm for considerations in the medical management (Fig. 2) of this often difficult‐to‐treat condition.

## Author Roles

(1) Research project: A. Conception, B. Organization, C. Execution; (2) Statistical Analysis: A. Design, B. Execution, C. Review and Critique; (3) Manuscript Preparation: A. Writing of the first draft, B. Review and Critique.

A.J.B.: 1B, 1C, 2C, 3A, 3B.

H.C.: 1B, 1C, 2A, 2B, 2C, 3B.

S.J.: 1A, 1B, 1C, 3A, 3B.

G.P.: 1A, 1B, 1C, 2C, 3B.

A.J.: 1A, 1B, 1C, 2C, 3B.

## Disclosures

**Ethical Compliance Statement:** Institutional review board ethical approval was not sought for this project. Informed patient consent was not necessary for this work. We confirm that we have read the Journal's position on issues involved in ethical publication and affirm that this work is consistent with those guidelines.

**Funding Sources and Conflict of Interest:** The authors declare that there are no funding sources or conflicts of interest relevant to this work.

**Financial Disclosures From The Last 12 Months:** AJB: There are no additional disclosures to report. HC: there are no additional disclosures to report. GP: There are no additional disclosures to report. SJ has received honoraria from Abbvie, Bial, Merz, UCB, Italfarmaco, Orion and Zambon. She holds a permanent position by Servicio Andaluz de Salud. She received grants from the Spanish Ministry of Economy and Competitiveness (PI18/01898) and the Consejería de Salud de la Junta de Andalucía (PI‐0459‐2018). AJ has received support to attend scientific conferences from Merz and research funding from Novartis. AJ is currently funded by the Wellcome Trust and the National Institute of Health Research University College London Hospitals Biomedical Research Centre.

## Supporting information


**Data S1.** Meta‐analysis method description.

## Data Availability

The data that support the findings of this study are available from the corresponding author upon reasonable request.
