# Supplementary material for: Management of Anxiety in Parkinson's Disease
Source: Mov Disord Clin Pract. 2025 Jun 26;12(10):1490–501. doi: 10.1002/mdc3.70144 (PMC12528972; doi:10.1002/mdc3.70144)
Supplement: Supplementary file 1 — Data S1. Meta‐analysis method description. [file MDC3-12-1490-s001.docx]

**SUPPLEMENT 1**

**Meta-analysis method**

Data was extracted from all randomised controlled trials included in the following systematic reviews where a measure of anxiety symptom severity was reported^1–3^:

1. Mills KA, Greene MC, Dezube R, Goodson C, Karmarkar T, Pontone GM. Efficacy and tolerability of antidepressants in Parkinson’s disease: A systematic review and network meta-analysis. *International Journal of Geriatric Psychiatry*. 2018;33(4):642-651. doi:10.1002/gps.4834

2. Starkstein SE, Brockman S. Management of Depression in Parkinson’s Disease: A Systematic Review. *Mov Disord Clin Pract*. 2017;4(4):470-477. doi:10.1002/mdc3.12507

3. Skapinakis P, Bakola E, Salanti G, Lewis G, Kyritsis AP, Mavreas V. Efficacy and acceptability of selective serotonin reuptake inhibitors for the treatment of depression in Parkinson’s disease: a systematic review and meta-analysis of randomized controlled trials. *BMC Neurol*. 2010;10:49. doi:10.1186/1471-2377-10-49

For each included trial, standardized mean differences (SMD) in anxiety symptom scores between treatment and control groups were calculated. The following formulae were used to convert study measures into SMD (or Cohen’s ds) and associated variances between subjects:

$$d= \frac{M_{1}-M_{2}}{{SD}_{pooled}}$$

**Equation 1.** Cohen’s d from Means and Standard Deviations of 2 samples. d is Cohen’s d, M1 is the mean of one sample M2 is the mean of the other sample, SDpooled is the pooled standard deviation of the two samples (please see below.)

$${SD}_{pooled}=\sqrt{{(N_{1}-1)SD}_{1}^{2}+ {(N_{2}-1)SD}_{2}^{2}}$$

**Equation 2.** Pooled standard deviation of 2 samples. SD pooled is the pooled standard deviation of the two samples, N1 is the size of one sample N2 is the size of the other sample, SD1 is the standard deviation of one sample, SD2 is the standard deviation of the other sample.

When multiple anxiety scales were reported within a study, we prioritised the most commonly used validated scale across studies.

Meta-analysis was performed using the R statistical programming language and the packages metafor and metaviz.

A random-effects model was used to pool effect sizes, accounting for variability between studies. The restricted maximum likelihood (REML) estimator was used for variance estimation, as it provides an unbiased estimate of between-study heterogeneity. Given that some included studies had multiple treatment arms, a multilevel meta-analysis model (rma.mv() function in metafor) was implemented to account for within-study correlations and ensure appropriate handling of dependency structures.

Heterogeneity was assessed using multiple approaches, including variance components and the Cochran’s Q statistic. The between-study variance component (σ²) was estimated to determine the degree of heterogeneity attributable to differences between studies. The Q-test for heterogeneity was performed to assess whether the observed variability in effect sizes exceeded what would be expected by chance, providing a statistical measure of inconsistency across studies.
